# Comparison of prevalence, viral load, physical status and expression of human papillomavirus-16, -18 and -58 in esophageal and cervical cancer: a case-control study

**DOI:** 10.1186/1471-2407-10-650

**Published:** 2010-11-26

**Authors:** Donghong Zhang, Qingying Zhang, Li Zhou, Leijun Huo, Yi Zhang, Zhongying Shen, Yi Zhu

**Affiliations:** 1 Cardiovascular Research Center, Shantou University Medical College, 22 Xinling Road, Shantou, Guangdong, 515041, China; 2 Department of Preventive Medicine, Shantou University Medical College, 22 Xinling Road, Shantou, Guangdong, 515041, China; 3 Department of Gynecologic Medical Oncology, Affiliated Cancer Hospital of Shantou University Medical College, 22 Xinling Road, Shantou, Guangdong, 515041, China; 4 Department of Physiology and Pathophysiology, Peking University Health Sciences Center, 38 Xueyuan Road, Beijing, 100191, China; 5 Institute of Oncology Pathology, Shantou University Medical College, 22 Xinling Road, Shantou, Guangdong, 515041, China

## Abstract

**Background:**

Human papillomavirus (HPV) infection is a major risk factor for the development of nearly all cases of cervical cancer worldwide. The presence of HPV DNA in cases of esophageal squamous-cell carcinoma (ESCC) has been reported repeatedly from Shantou, China, and other regions with a high incidence of esophageal carcinoma (EC). However, unlike in cervical squamous-cell carcinoma (CSCC), in ESCC, the characteristics of HPV are unclear. Thus, the role of high-risk HPV types in the carcinogenesis of ESCC remains uncertain.

**Methods:**

Seventy cases of ESCC with 60 controls and 39 cases of CSCC with 54 controls collected from patients in Shantou region in China were compared for the distributions of HPV-16, -18 and -58; viral load; and viral integration using real-time PCR assay and HPV-16 expression using immunostaining.

**Results:**

The detection rates and viral loads of HR-HPV infection were significantly lower in ESCC than in CSCC (50.0% vs. 79.48%, P = 0.005; 2.55 ± 3.19 vs. 361.29 ± 441.75, P = 0.002, respectively). The combined integration level of HPV-16, -18 and -58 was slightly lower in ESCC than in CSCC (P = 0.022). HPV-16 expression was detected in 59.26% of ESCC tissue and significantly associated with tumour grade (P = 0.027).

**Conclusions:**

High levels of HR-HPV expression and integration may be an indicator of the risk of ESCC, at least for patients in the Shantou region of China. However, a relatively low HPV copy number and infection rate in ESCC is unlikely to play an essential a role in the carcinogenesis of ESCC as in cervical cancer. Factors other than HR-HPV infection may contribute to the carcinogenesis of ESCC.

## Background

Esophageal squamous cell carcinoma (ESCC) occurs worldwide and has a variable geographic distribution [1]. The Shantou coastal area of eastern Guangdong province, in southern China, has a high incidence of esophageal carcinoma (EC) [2]. Tobacco smoking, alcohol drinking, dietary deficiency, and some local factors, including drinking hot tea and consumption of fermented fish sauce, may be involved in the development of EC in this region [3,4]. However, the etiology of EC is still largely unknown.

The association of HPV infection and EC has been reported in the last 30 years, especially in geographic areas with a high incidence of EC. However, unlike cervical carcinoma, with its almost conclusive association with HPV, the causal role of HPV infection in EC remains controversial. This lack of association is due in part to the wide variation in reported infection rates among different studies (from 0% to more than 60%), and few studies have related HPV status to genetic changes [5]. These variations may reflect a difference in HPV infection rates in EC cases collected from different geographic regions. However, differences in sensitivity and specificity of the detection methods may also be contributors. Previously, we found a high prevalence of HPV infection in esophageal cancerous (65.5%) and para-cancerous (69.1%) tissues and matched normal esophagus (60.0%) tissues in the Shantou region by a regular PCR method [6]. In addition, our *in vitro *experiments indicated that expression of the HPV-18 E6/7 gene was sufficient to induce cellular immortality, including cellular proliferation, telomerase activity and chromosome instability [7].

More than 40 genotypes of HPV that infect the anogenital area are associated with a large spectrum of diseases from benign proliferative to invasive cancers. In China, a meta-analysis revealed that high percentages of cervical cancer cases mainly attributed to the 3 most frequent high-risk types of HPV (HR-HPV): HPV-16 is involved in more than half of the cervical cancer cases, and HPV-18 and -58, approximately 11.0% and 7.2%, respectively [8]. Recent studies have suggested a high correlation between HR-HPV viral load (notably HPV-16) and high-grade squamous-cell intraepithelial lesions [9,10]. In addition, the disruption of the HPV E2 gene during viral integration, which results in a loss of its function as a regulator of viral oncogene expression and subsequent upregulation of E6/E7 gene transcription, may initiate cell transformation and immortality by deregulating the tumor suppressor gene products p53 and retinoblastoma protein [11]. Thus, both HR-HPV DNA load and its integration may be 2 candidate markers that could help identify high risk of progression of cervical intraepithelial neoplasms and forecast cervical lesions in patients with chronic infection. In addition, despite multiple investigations based on various study designs and methodologies, the clinical utility of HR-HPV DNA load remains unclear [12].

Only one study detailing the characteristics of HR-HPV DNA load in ESCC from the Shantou region has been reported; median copy numbers (11.7-14.6 copies/genome equivalents) were relatively higher than that in other regions in China, with frequent integration of HPV-16 DNA in the host genome. In this study, only four HPV-16-positive ESCC cases were detected [13]. Thus, a discrepancy in findings is inevitable considering the relatively small number of samples. We aimed to characterize the prevalence, viral load and physical status of the 3 most common HPV types, HR-HPV-16, -18 and - 58, in ESCC and CSCC and their corresponding control tissues. We used a real-time PCR-based method for measurement of HPV-16, -18 and -58 viral load and physical status in clinical samples. This method is accurate and sensitive for detecting as few as 50 copies of integrated HPV DNA in clinical samples [14]. We hoped to increase our knowledge of HR-HPV-induced esophageal and cervical carcinogenesis. Further, HPV-16 expression was detected by immunohistochemistry (IHC) to evaluate its role in ESCC tissue.

## Methods

### Subjects and Study Design

The study was carried out in compliance with the Declaration of Helsinki and was approved by the Ethics Committee of Shantou University Medical College. All subjects gave their written informed consent to participate in the study (Department of Medical Oncology, Affiliated Cancer Hospital of Shantou University Medical College, China).

Fresh ESCC tissue was from 70 patients with ESCC (49 males; mean age 57.32 ± 8.44 years; 21 females; mean age 59.95 ± 8.77 years) and 60 subjects with normal esophageal tissue (38 males; mean age 31.73 ± 8.62 years; 22 females; mean age 45.91 ± 5.72 years) from the Department of Thoracic Surgery in the Affiliated Tumor Hospital of Shantou University Medical College from 2002 to 2006. Control samples were from post-mortem paraffin-embedded esophageal tissue without lesions of subjects who died due to trauma (in the hospital or daily life [mainly traffic accidents and regular daily labor]) or cardiocerebrovascular disease. Fresh CSCC tissue was from 39 patients with CSCC (mean age 43.10 ± 8.16 years) and 54 patients with normal cervical tissue (mean age 44.14 ± 8.27 years) from the Department of Gynecological Tumors of the Affiliated Tumor Hospital of Shantou University Medical College from 2006 to 2008. We collected 54 paraffin-embedded samples from each of 54 ESCC patients (mean age 52.52 ± 9.89 years) from the Affiliated Tumor Hospital of Shantou University Medical College from 1991 to 1995. All subjects had lived or were living in the Shantou region.

Hematoxylin and eosin-stained tissue sections from ESCC, CSCC, and corresponding control tissue were reviewed by Professor Shen Zhongying from the Institute of Oncology Pathology to confirm the original findings and define representative tumor regions. Tumor stage and grade were defined according to the World Health Organization histological classification criteria [15].

### HPV plasmid construction

pMD™ 18-T Simple Vector (Takara Biotechnology [DALIAN] Co., Japan) plasmids containing E2 and E6/7 genes for HPV-16, -18, -58 were prepared by cloning their corresponding PCR products from clinical samples. The inserted fragments were confirmed by sequencing and PCR amplification. The plasmids were used as positive controls. PCR primer sequences are in Table 1.

**Table 1:** PCR primer sequences for human papillomavirus (HPV) and β-globin

Gene	Primer	Primer Sequence (5'→3')	Product	**AT**^**‡ **^**(°C)**
HPV-16 E6/7	F*	GAATGTGTGTACTGCAAGCA	195 bp	56
	R^†^	GTTGTATTGCTGTTCTAAGTTGT		
HPV-16 E2	F	ACTGTGGTAGAGGGTCA	177 bp	56
	R	TTCGTTGCTGCTAAA		
HPV-18 E6/7	F	GTATGGACCTAAGGCAACA	155 bp	52
	R	GTCGGGCTGGCAAA		
HPV-18 E2	F	ACGGTATCCGCTACTCAG	140 bp	52
	R	GTCTCCGCAAGTCCA		
HPV-58 E6/7	F	ATGTGACAGCTCAGACGAGG	166 bp	53
	R	CAGCTGCTGTAGGGTTCGT		
HPV-58 E2	F	GTGGGTAGTCGGGTAA	129 bp	53
	R	TCGTCGTCGCTTTG		
β-globin	F	AACAGCATCAGGAGTGGAC	102 bp	52
	R	CTGCCTATTGGTCTATTTTCC		

*Forward.

^†^Reverse.

^‡^Annealing Temperature.

### Real-time PCR for HR-HPV E2 and E6/7

Fresh samples from the ESCC and CSCC cases and their controls were used for HPV detection and genotyping with high-sensitive real-time PCR. The plasmid stock solutions were diluted in sterile water to obtain a quantification gradient of 10^10^, 10^8^, 10^6^, 10^4^, 10^2 ^and 1 copy. Real-time PCR reactions involved use of an ABI Prism^® ^7300 system. The 25-μl reaction mixture included SYBR^® ^*Premix Ex Taq*™ (Takara Biotechnology [DALIAN] Co.); forward and reverse primers at 10 pmol/ml for HPV-16, -18 and -58 E2 or E6/7 genes; and 2 μl DNA sample. PCR reactions were as follows: 50°C for 2 min, denaturing at 95°C for 5 min, then 40 cycles of 95°C for 15 sec and 60°C for 31 sec. Positive (corresponding plasmid) and negative (no DNA template) controls were introduced systematically in each reaction plate. Each sample was tested in duplicate. All experiments were repeated and confirmed separately by use of the Mx3000P™Real-Time PCR System (Agilent, Santa Clara, CA, USA).

### Validation of real-time PCR for HPV load and physical status

**Figure 1:**
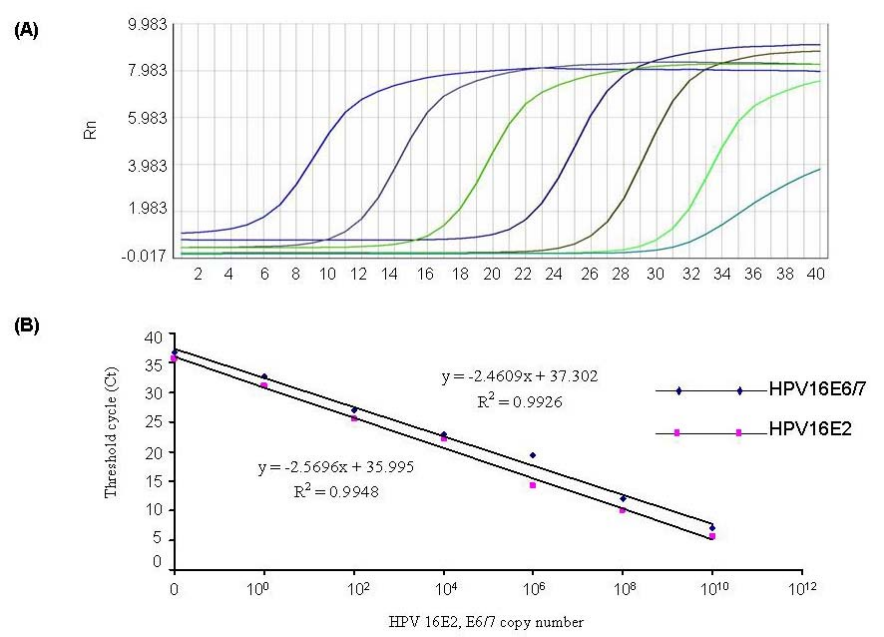
**Real-time PCR for human papillomavirus (HPV) DNA load and physical status assay**. (a) Typical amplification plots obtained by real-time PCR for 7-point series concentration of HPV-16 E6/7 plasmid DNA. The x axis denotes the cycle number and y axis the fluorescence intensity over the background. (b) Comparison of standard curves for the crossing points plotted against the HPV-16 E2 and E6/7 concentrations. The x axis denotes the cycle number and y axis the series concentration of HPV-16 plasmid DNA. Data are mean ± SE (n = 3).

The detection limit of the real-time PCR method was determined by using 100-fold gradient dilutions of input plasmid DNA. All typical amplification plots involved each HPV type for E2 and E6/7 plasmid DNA (change in the fluorescent signal versus cycle number). HPV-16 E6/7 plasmid DNA amplification plots are in Figure 1a: the crossing points for the real-time PCR ranged from 10^10 ^to 1 copy for HPV-16 E6/E7 DNA (dilution factor 100) when present in amounts from 1 ng to 0.1 fg. Hence, we were able to detect HPV at amounts as low as 0.1 fg, equivalent to 1 copy. The standard curve showed a dynamic linear range for quantification across copy number over the entire range of dilution concentrations on bivariate correlation analysis (R^2 ^= 0.9926) (Figure 1b). Similar results were obtained with amplification plots for E2 and E6/7 genes of HPV-16, -18 and -58 and the β-globin standard (data not shown).

Because HPV-16 integration often disrupts the E2 gene, recent assays measuring HPV-16 integration were based on quantification with real-time PCR of HPV-16 E6/7 relative to E2 [16]. As previous study proposed, assignment of integrated, mixed or episomal physical status of HPV DNA was calculated for each clinical specimen and determined by measuring the ratio of E2 to E6/7 [17]. The protocol for the assay for ratio of E2 to E6/7 was modified to include dual standard curves of E2 and E6/7. Table 2 shows the standards for determining the HPV-16, -18 and -58 DNA physical statuses with ratios of E2 to E6/7. Each ratio of E2 to E6/7 was calculated from the same respective template. The ratio cut-off (mean ± SE) of E2 to E6/7 loads for each HPV type were calculated by E2 and E6/7 gene amplification with use of the template made with a mixture of equivalent HPV E2 and E6/7 plasmids (range from 10^10 ^to 1 copy) representing the episomal-form control and with the gradient dilution-constructed HPV E6/7 plasmids representing the integrated-form control. The integration form was defined by the absence of the E2 signal or ratio <0.001. Ratios between 0.001 and the lower episomal ratios indicated the mixed form (presence of both integrated and episomal forms).

**Table 2:** Standards to judge HPV physical status by ratio of E2 to E6/7 gene

Viral	E2:E6/7 ratio		
	
	Integration	Mixed	Episomal
HPV-16	<0.001	0.001-0.864	>0.864 (1.005 ± 0.141)
HPV-18	<0.001	0.001-0.823	>0.823 (1.004 ± 0.181)
HPV-58	<0.001	0.001-0.780	>0.780 (0.892 ± 0.112)

### Immunohistochemistry

Paraffin-embedded samples from the 54 additional ESCC cases were used to create a tissue microarray (TMA) block for IHC detection of HPV-16 expression. Streptavidin-peroxidase (SP) IHC staining was performed according to the manufacturer's instructions for the SP kit (Maxim.bio Co., China) and was as described previously [18]. In brief, slides were deparaffinized in xylene, and endogenous peroxidase was inactivated by immersing the slides in a 0.3% H_2_O_2_-methanol solution for 30 min at room temperature. Serum blocking solution (100 μl) was added to each section for 10-min incubation, and then anti-HPV-16 monoclonal antibody (Maxim.bio Co.) was added for 30 min. After a rinsing, SP-conjugated polymer (100 μl) was added to each section for 10 min. Sections were stained with 100 μl DAB chromogen (Maxim.bio Co.) and restained with haematoxylin for visualization of nuclei for 3 to 10 min. After a rinsing, slides were counterstained with Mayer's hematoxylin, dehydrated, and mounted. Paraffin sections of cervical carcinoma served as positive controls. For negative controls, primary antibodies were substituted with phosphate buffered saline instead of HPV-16 antibody.

**Figure 2:**
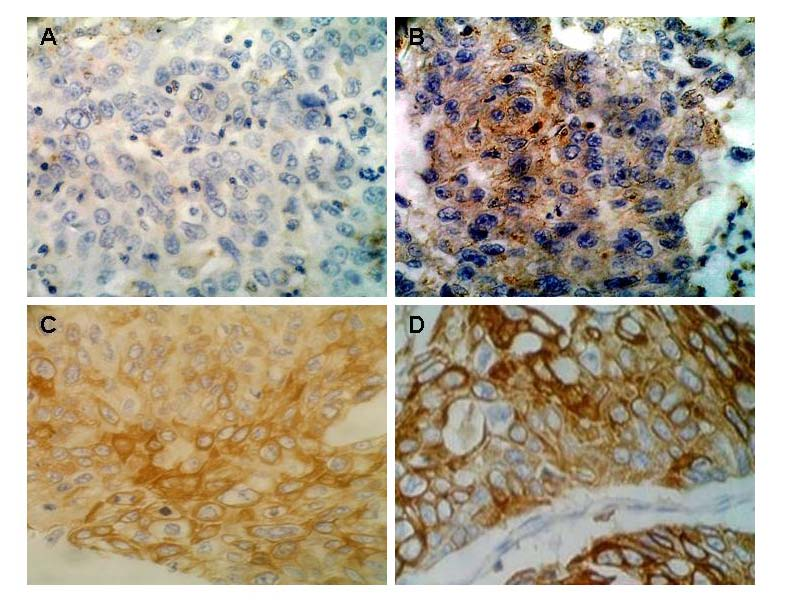
**Immunohistochemistry results targeting HPV-16 in tumor samples (cancer grade I-III)**. The positive expression of HPV-16, indicated by brown immunohistochemistry signals, located mainly in the cytoplasm of cancer cells. A, negative (-) in cancer grade **I **sample; B, slightly positive (±) in cancer grade **II **sample; C, positive (+) in cancer grade **II **sample; D, strongly positive (++) in cancer grade **III **sample. The brown staining was by DAB and the nuclei were slightly stained by haematoxylin (original magnification × 200).

Positive staining for IHC was as described [19] with modification. Briefly, staining for HPV-16 was mainly in cytoplasm. The immunoreactivity for all proteins was defined as follows: strongly positive (++), more than 50% of tumor cells stained; positive (+), more than 25% of the cells stained; slightly positive (±), more than 10% of the tumor cells stained; and negative (-), less than 10% of the tumor cells stained or tumor cells lacking immunoreactivity (Figure 2). All slides were observed by 2 independent pathologists in a double-blinded method, and the final concordant results were adopted.

### Statistical analysis

The presence of HPV DNA among different groups was analyzed by chi-square test. HPV viral load, physical status and HPV-16 expression were analyzed by cancer type, age, lymph-node metastasis and cancer grade by independent-samples *t *test, one-way ANOVA, chi-square test, Kruskal-Wallis assay, Mann-Whitney-U, Fisher exact test or Spearman correlation analysis as appropriate. The level of statistical significance was set at 0.05 (2-sided). All analyses involved use of SPSS v16.0 (SPSS Inc., Chicago, IL).

## Results

### Prevalence, viral loads and physical status of HPV-16, -18 and -58

The detection rates for total infection with HPV-16, -18 and -58 DNA in ESCC or CSCC tissue were significantly higher than those of their corresponding controls: 50.00% vs. 33.33% for ESCC, P = 0.045; and 79.48% vs. 18.52% for CSCC, P < 0.001. Patients with CSCC showed a higher total detection rate than those with ESCC (P = 0.005). HPV-16 was the most prevalent type of single infection in all groups, followed by HPV-58 and -18 (Table 3).

**Table 3:** HPV-16, -18 and -58 prevalence, viral load and physical status in patients with ESCC and CSCC, and in their corresponding controls

Origin	Total	No. (%)	Viral load (copies/cell)			Physical status (E2:E6/7 ratio)			
			
			Min-max	Mean ± SD	Mean ± SD	Episomal	Mixed		Integration
HPV16						>0.86	0. 10-0.86	0.001-0.10	<0.001
ESCC	70	21(30.00)	0.02-9.28	2.19 ± 2.60	0.25 ± 0.37	2	5	8	6
NE	60	13(21.67)	0.04-2.08	0.55 ± 0.16	0.51 ± 0.36	3	7	2	1
CSCC	39	19(48.72)	32.03-1546.38	468.39 ± 473.36	0.03 ± 0.08	0	5	5	9
NC	54	8(14.81)	0.12-122.92	26.21 ± 34.26	0.59 ± 0.44	4	3	1	0
HPV18						>0.82	0. 10-0.82	0.00-0.10	<0.001
ESCC	70	8(11.43)	0.00-11.29	2.03 ± 3.88	0.17 ± 0.14	0	1	6	1
NE	60	3(2.00)	0.03-2.25	0.73 ± 1.26	0.37 ± 0.14	0	2	0	1
CSCC	39	7(17.95)	5.14-295.38	174.77 ± 121.20	0.06 ± 0.10	0	2	3	2
NC	54	2(3.70)	1.22-130.51	65.87 ± 91.42	0.60 ± 0.34	1	1	0	0
HPV58						>0.78	0. 10-0.78	0.001-0.10	<0.001
ESCC	70	9(21.43)	0.03-12.56	3.86 ± 3.82	0.17 ± 0.32	1	1	3	4
NE	60	4(6.67)	0.17-2.65	1.68 ± 1.09	0.35 ± 0.31	0	3	1	0
CSCC	39	9(23.08)	4.11-1521.70	280.27 ± 499.13	0.12 ± 0.18	0	3	0	6
NC	54	2(3.70)	0.99-106.59	53.79 ± 74.67	0.64 ± 0.26	1	1	0	0
3 HR-HPVs									
ESCC	70	35(50.00)	0.00-12.56	2.55 ± 3.19 ***^a^***	0.20 ± 0.32	3	6	16	10***^d^***
NE	60	20(33.33)	0.04-2.08	0.55 ± 0.57	0.53 ± 0.33	3	12	3	2
CSCC	39	31(79.48)	4.11-1546.38	361.29 ± 441.75***^bc^***	0.09 ± 0.14	0	9	6	16***^e f^***
NC	54	10(18.52)	0.12-853.43	30.87 ± 57.39	0.55 ± 0.40	6	3	1	0

*^a^Z *= -2.594, P = 0.029, compared with NE group; *^b ^Z *= -3.115, P = 0.002 and *^c^Z *= -4.827, P < 0.001, compared with ESCC patients and NC group, respectively, and calculated with the Mann-Whitney-U test.

*^d ^***χ^2 ^**= 12.703, P = 0.004; *^e ^***χ^2 ^**= 22.544, P < 0.001; *^f ^***χ^2 ^**= 8.850, P = 0.022 compared with NE group. NC group and ESCC patients, respectively, and calculated with the Chi-square test or Fisher exact test as appropriate.

ESCC, esophageal squamous cell carcinoma; CSCC, cervical squamous cell carcinoma; NE, normal esophagus; NC, normal cervix.

The overall viral loads of HPV-16, -18 and -58 infection are listed in Table 3. Our data showed no significant difference in mean viral loads of HPV-16, -18 and -58 within each group. However, the combined viral load for all HR-HPVs in ESCC and CSCC patients was significantly higher than that of their corresponding controls: ESCC, 2.55 ± 3.19 vs. 0.55 ± 0.57 copies/cell (P = 0.029); and CSCC, 361.29 ± 441.75 vs. 30.87 ± 57.39 copies/cell (P < 0.001). In addition, the mean viral load of HPV-16 in CSCC patients was approximately 141.68-fold more than that in ESCC patients (468.39 ± 473.36 vs. 2.55 ± 3.19 copies/cells, P = 0.002).

In the present study, we calculated a mean ratio of E2 to E6/7 for each group to determine the degree of viral DNA integration (Table 3). Patients with CSCC showed a significantly lower mean ratio of E2 to E6/7 for HPV-16, -18 and -58 than that of normal controls (0.09 ± 0.14 *vs*. 0.55 ± 0.40, P < 0.001), which indicates a high prevalence of the DNA integration form of HPV in CSCC patients. However, ESCC patients and controls did not differ in mean ratio of E2 to E6/7 for HPV-16, -18 and -58 (0.20 ± 0.32 vs. 0.53 ± 0.33, P = 0.06, also see Table 3). Moreover, for CSCC patients, the mean ratios of E2 to E6/7 for HPV-16, -18 and -58 were 0.03 ± 0.08, 0.06 ± 0.10 and 0.12 ± 0.18, respectively, with no significant difference in HPV ratios (P = 0.229); similar results were found for ESCC patients and controls and CSCC controls.

Furthermore, results for HR-HPV physical status showed DNA episomal, integrated and mixed forms (Table 2). HPV DNA integration and mixed forms were more frequently observed in CSCC patients (100% [31/31]) than in controls (40.00% [4/10]) (P < 0.001). The frequency of HR-HPV integration markably increased with CSCC grade (*r_s _*= 0.575, P < 0.001). Similarly, for ESCC patients, the frequency of HR-HPV integration was higher than that for ESCC controls (*r_s _*= 0.405, P = 0.002). Overall, the mixed and integrated forms of HPV were commonly observed in ESCC patients and controls (91.43% [32/35] *vs*. 85.00% [17/20]).

### Relationship between HPV DNA infection and clinical characteristics of patients with ESCC or CSCC

We analyzed whether the clinical and histological characteristics of patients with ESCC and CSCC were associated with HPV infection. For CSCC patients, HR-HPV loads were significantly increased and ratios of E2 to E6/7 were decreased with increased tumour grade of CSCC (P = 0.008 and P = 0.042, respectively) (Table 4). For ESCC patients, the integration level was associated with pathological grade of ESCC (P = 0.015). However, no significant associations were found between HPV physical status and patient age or lymph-node metastasis for CSCC patients. HPV viral loads were not associated with ESCC patient age, lymph-node metastasis or cancer grade (Table 4).

**Table 4:** Relation of viral load and ratio of E2 to E6/7 of HP-HPV infection and clinical characteristics of ESCC and CSCC patients

Characteristics	No. of subjects	HPV viral load (mean ± SE)	*t/F/Z *statistic	P*	HPV E2:E6/7 ratio (mean ± SE)	*t/F/Z */statistic	P*
**ESCC patients**							
Age (years)							
54.42 ± 7.86	38	2.55 ± 3.19	-0.152	0.364^#^	0.201 ± 0.325	0.049	0.771^#^
Lymph node metastasis							
Yes	20	2.98 ± 3.81	0.879	0.385	0.22 ± 0.30	-0.731	0.478
No	18	2.07 ± 2.34			0.18 ± 0.34		
Cancer grade							
I	13	1.65 ± 3.42			0.39 ± 0.43		
II	20	2.96 ± 3.32	0.799	0.458	0.12 ± 0.23	8.361	**0.015**
III	5	3.24 ± 1.60			0.05 ± 0.11		
**CSCC patients**							
Age (years)							
43.42 ± 8.44	35	361.29 ± 441.74	-0.108	0.537^#^	0.09 ± 0.14	-0.184	0.289^#^
Lymph node metastasis							
Yes	10	330.03 ± 316.40	-0.110	0.913	0.11 ± 0.12	-1.278	0.201
No	25	373.80 ± 448.21			0.08 ± 0.16		
Cancer grade							
I	14	188.68 ± 374.64			0.17 ± 0.19		
II	12	340.28 ± 262.40	9.542	**0.008**	0.06 ± 0.09	6.363	**0.042**
III	9	660.97 ± 592.94			0.007 ± 0.001		

*P values for the differences of HPV viral load and ratio of E2 to E6/7 among different groups was calculated with the independent-samples *t *test or Mann-Whitney-U test, one-way ANOVA or Kruskal-Wallis assay as appropriate.

^# ^P values for the correlation between the age of patients and HPV viral load, as well as HPV ratios of E2 to E6/7 were calculated by Spearman correlation.

ESCC, esophageal squamous cell carcinoma; CSCC, cervical squamous cell carcinoma.

### Relationship between HPV-16 expression and clinical characteristics of patients with ESCC

To determine whether the expression of HPV-16 might have an influence on esophageal malignant progression, we analyzed HPV-16 expression by clinical characteristics of patients with ESCC. As shown in Table 5, HPV-16 expression was detected in 59.26% of ESCC tissues and was positively, although weakly, correlated with pathological grade of cancer and age of ESCC patients (r_s _= 0.301, P = 0.027; r_s _= 0.229, P = 0.028, respectively). No relationship was found with lymph-node metastasis.

**Table 5:** Correlation between HPV-16 expression and clinical characteristics of ESCC patients

Characteristics	No.	HPV-16 *				*r*	***P***^***#***^
				
		-	±	+	++		
Age (years)							
53.13 ± 10.05	54	22	12	15	5	0.299	**0.028**
Lymph-node metastasis							
Yes	36	16	8	9	3	0.115	0.408
No	18	6	4	6	2		
Cancer grade							
I	16	8	3	2	1	0.301	**0.027**
II	28	13	8	8	3		
III	10	1	1	5	1		

* indicates positive on immunohistochemistry staining.

*^# ^*P values for the correlation between HPV-16 expression and clinical characteristics of ESCC patients were calculated by Spearman correlation or Fisher exact test as appropriate.

## Discussion

We adopted a comprehensive methodology to assess the prevalence, viral load and physical status of infection with HR-HPV-16, -18 and -58, the 3 most common HR-HPVs, in patients with ESCC and CSCC and the expression of HPV-16 in patients with ESCC. We aimed to explore the characteristics of HR-HPV DNA in the carcinogenesis of ESCC compared with CSCC.

Infection with the 3 types of HR-HPV associated with CSCC (79.48%) in the Shantou region was consistent with that in other regions in China, but the infection in controls was higher (18.52% vs. 6.87%) [8,20]. This increased rate should be interpreted with caution because of the small number of cases in our study. For ESCC patients, the total infection rate for all 3 viruses was greater than that of their controls. Among the HR-HPV types, HPV-16 was the most predominant in all esophageal tissues. Our results for HPV-16 and -18 infection rates in Shantou were similar to those from a previous study (52.4%) [21] and were higher than those in other regions [22,23]. Interestingly, this distribution of HR-HPV in esophageal lesions was in agreement with observations for cervical lesions [24].

Over the past 20 years, reported HPV infection rates have varied in ESCC patients from geographically different areas [5], but are consistently high in CSCC patients [25]. This significant difference may result from HPV DNA copy numbers in host tissues. Evidence from our study revealed that the average viral load for HR-HPV for CSCC patients was significantly higher than that for ESCC patients in Shantou region; the mean viral load for HPV-16 was 2.55 ± 3.19 copies/cell in ESCC tissues. This low figure was also found in a Chinese study of EC tissues (<1 to 157 copies/cell) [13,26] and a Finnish study of head and neck SCCs (4.6 to 49 copies/cell) [27]. Extremely low levels of HPV DNA load (even 0.01 copies/cells) in ESCC tissues may exceed the detection ability of some techniques such as regular PCR and conventional *in situ *hybridization (ISH), which limits detection to <10-50 copies/cell [28]. Our positive results for <10 copies of HPV DNA would be out of the detection range with use of general PCR and ISH technology. This low detection rate might explain why we failed to measure the level of HPV mRNA in our paraffin-embedded tissue by RT-PCR and the low detection rates we found with regular PCR in our previous study. This low HPV infection load was not correlated with clinical characteristics such as age, cancer grade or lymph-node metastasis of ESCC patients and their controls. However, increased levels of all HR-HPVs with increasing cancerous grade indicated a dose-dependent association of viral load and cancer grade.

Epidemiologic and molecular studies have shown that HR-HPV DNA integration is considered a prerequisite for the development of several malignant lesions [29,30]. For the integration level of viral DNA, we found no differences between CSCC and ESCC lesions for the level of integration of HPV-16, -18 and -58; most infected CSCC or ESCC control subjects carried the episomal form of the virus, whereas the mixed and integrated forms were predominant in ESCC and invasive cervical carcinomas. For ESCC patients, specifically, we found both ratios of HPV DNA integration and expression of HPV-16 protein increased with ESCC grade. The integration occurs in the early stage of esophageal carcinogenesis and was associated with the severity of the cancer. So the increased expression of the E6 and E7 oncoproteins in ESCC might result from disrupting the E2 open reading frame when HPV integrates into the host genome [31-33]. Although the direct evidence of HPV-16 transcription and expression derived from integrated HPV DNA is still unclear, from previous studies, HPV E6- and E7-encoding cDNAs derived from integrated viral oncogene transcripts have a much stronger transforming capacity in primary cells than cDNAs derived from episome-derived transcripts [11]. The relative expression levels of the viral oncogenes and their corresponding gene products appear to be directly influenced by the sequence context of individual integration sites in cervix, head, neck, penile and tonsil carcinogenesis [34,35]. So the potential connections among HPV DNA integration, transcription and expression, as well as its clinical value, need to be confirmed with more studies.

Our data confirmed for CSCC that the high prevalence, viral loads and integration rates of HR-HPV were the most important risk factors for cervical carcinogenesis. We also found relatively low HPV-16, -18 and -58 viral load in ESCC patients, which is consistent with the relatively low HR-HPV DNA viral load found in some cervical carcinoma cell lines such as SiHa cells (1-2 copies/cell for HPV-16) and HeLa cells (10-50 copies/cell for HPV-18). However, the low viral load, particularly with a common integrated viral genome and high expression of its onco-protein, may be sufficient to lead to or promote carcinogenesis, especially because of the high incidence of EC.

Our study contains some limitations. The selection bias cannot be avoided because of the relatively small number of samples collected, which may reduce the representativeness of our results and capacity for inference. Heterogeneity between ESCC and CSCC patient ages also existed because of the small number of samples in these groups.

## Conclusions

Our data show that a relatively low HPV copy number and infection rate in ESCC is unlikely to play an essential a role in the carcinogenesis of ESCC as in cervical cancer. However, the presentation of the physical status and expression of the HR-HPV types in ESCC strongly suggests a possible role of oncogenic HPV infection in carcinogenesis of ESCC, at least for patients in the Shantou region of China. Because esophageal carcinogenesis is a complex, multi-step process, additional work is warranted to elucidate the underlying molecular mechanisms and the genetic changes associated with HPV infection in the development of esophageal cancer.

## Competing interests

The authors declare that they have no competing interests.

## Authors' contributions

DZ, LH and YZha carried out HPV detection and typing. DZ and ZS participated in HPV-16 protein detection. LZ participated in sample preparation and data validation. QZ performed the statistical analysis. DZ, ZS, and YZhu participated in study design and coordination, and prepared the manuscript. All authors read and approved the final manuscript

## Pre-publication history

The pre-publication history for this paper can be accessed here:

http://www.biomedcentral.com/1471-2407/10/650/prepub
